# Structural and Optical Properties of Silicon Nanowire Arrays Fabricated by Metal Assisted Chemical Etching With Ammonium Fluoride

**DOI:** 10.3389/fchem.2018.00653

**Published:** 2019-01-04

**Authors:** Kirill A. Gonchar, Veronika Y. Kitaeva, George A. Zharik, Andrei A. Eliseev, Liubov A. Osminkina

**Affiliations:** ^1^Physics Department, Lomonosov Moscow State University, Moscow, Russia; ^2^Chemistry Department, Lomonosov Moscow State University, Moscow, Russia; ^3^Faculty of Materials Science, Lomonosov Moscow State University, Moscow, Russia; ^4^Institute for Biological Instrumentation of Russian Academy of Sciences, Pushchino, Russia

**Keywords:** silicon nanowires, impedance, total reflectance, photoluminescence, Raman scattering

## Abstract

Here we report on the metal assisted chemical etching method of silicon nanowires (SiNWs) manufacturing, where the commonly used hydrofluoric acid (HF) has been successfully replaced with ammonium fluoride (NH_4_F). The mechanism of the etching process and the effect of the pH values of H_2_O_2_: NH_4_F solutions on the structural and optical properties of nanowires were studied in detail. By an impedance and Mott-Schottky measurements it was shown that silver-assisted chemical etching of silicon can be attributed to a facilitated charge carriers transport through Si/SiO_x_/Ag interface. It was shown that the shape of nanowires changes from pyramidal to vertical with pH decreasing. Also it was established that the length of SiNW arrays non-linearly depends on the pH for the etching time of 10 min. A strong decrease of the total reflectance to 5–10% was shown for all the studied samples at the wavelength <800 nm, in comparison with crystalline silicon substrate (c-Si). At the same time, the intensities of the interband photoluminescence and the Raman scattering of SiNWs are increased strongly in compare to c-Si value, and also they were depended on both the length and the shape of SiNW: the biggest values were for the long pyramidal nanowires. That can be explained by a strong light scattering and partial light localization in SiNWs. Hereby, arrays of SiNWs, obtained by using weakly toxic ammonium fluoride, have great potential for usage in photovoltaics, photonics, and sensorics.

## Introduction

In recent decades, the possibility of using silicon nanowires (SiNWs) in sensorics (Cui et al., 2001; Wang and Ozkan, 2008; Cao et al., 2015; Georgobiani et al., 2018), photovoltaics (Kelzenberg et al., 2008; Stelzner et al., 2008; Sivakov et al., 2009), photonics (Brönstrup et al., 2010), and micro-and optoelectronics (Föll et al., 2010; Yang et al., 2010) has been shown. Nanowires are usually obtained as a result of anisotropic growth of a 1D crystal on a nanometer scale. The first SiNWs were fabricated via bottom-up approach by vapor-liquid-solid (VLS) method with different noble metals (mostly gold) as catalyst (Wagner and Ellis, 1964). Metal-assisted chemical etching (MACE) of silicon was observed for the first time in the 1990s, when a cleaning solution HF-H_2_O_2_-H_2_O was used to remove metal impurities from the silicon substrate (c-Si) (Morinaga et al., 1995). Then this method was adapted for luminescent porous silicon formation (Gorostiza et al., 1999; Li and Bohn, 2000; Chattopadhyay et al., 2002). In 2002, Peng et al for the first time adapted it for high aspect ratio SiNWs fabrication and systematically investigated the mechanism and further develop it into a new mciro/nanofabrication method (Peng et al., 2002, 2006, 2008). Also MACE method of SiNWs fabrication was systematically investigated in Nahidi and Kolasinski (2006), Sivakov et al. (2010), Bai et al. (2012), and Dawood et al. (2012). Usually in MACE such catalysts, as nanoparticles of Au (Li and Bohn, 2000; Dawood et al., 2012), Ag (Sivakov et al., 2010), or Pt (Li and Bohn, 2000; Chattopadhyay et al., 2002) and such oxidizing agents as H_2_O_2_ (Li and Bohn, 2000; Sivakov et al., 2010; Dawood et al., 2012), KMnO_4_ (Bai et al., 2012; Jiang et al., 2017), or Fe(NO_3_)_3_ (Nahidi and Kolasinski, 2006), are used in the process. SiNWs, which were fabricated by a standard MACE procedure, are found to possess such optical properties as extremely low total reflection (Gonchar et al., 2012), enhancement of Raman scattering and interband photoluminescence (PL) (Gonchar et al., 2014). However, HF, that is surely used in the MACE, is toxic and dangerous, and may also lead to hypocalcemia and hypomagnesemia (Bertolini, 1992). Therefore, it is very important, with a view to the future large-scale production of SiNWs, to study the possibilities of modifying the MACE method using safer and less toxic chemicals.

It is well-known that aqueous solutions of ammonium fluoride (NH_4_F) can be used to dissolve SiO_2_, and the etching rate depends on the concentration of NH_4_F and the pH of the solutions (Judge, 1971). Thus, NH_4_F is shown can be used as an alternative to HF in the method of electrochemical etching in the manufacture of porous silicon, and the structural properties of the resulting porous silicon depend on the pH of the NH_4_F solution used: at pH = 4.5 a pebble-like surface was formed, and at lower PH a nanoporous silicon layers were formed (Dittrich et al., 1995). Recently, the possibility of using NH_4_F in the MACE process has been also shown, and optical properties of SiNW, formed using NH_4_F, differed little from nanowires formed by standard MACE technology with HF (Gonchar et al., 2016). However, the mechanism of the etching process and the influence of the pH of the etching solution on the structural and optical properties of SiNW remain open.

In this work, the etching process mechanism and the effect of pH values of H_2_O_2_:NH_4_F solutions on the structural and optical properties of SiNWs were studied using impedance measurements and Mott-Schottky analysis, as well as total reflectance, interband photoluminescence and Raman scattering intensities measurements.

## Methods

The samples of SiNWs were produced by MACE of (100)-oriented p-type c-Si wafer with resistivity of 10–20 Ω•cm. HF was replacement on NH_4_F in all reactions. The PH value was controlled by PH indicator. Prior to the MACE procedure, c-Si wafers were rinsed in 2% HF solution for 1 min to remove a native silicon oxide. In the first stage of MACE process, c-Si wafers were placed in the aqueous solution of 0.02 M of silver nitrate (AgNO_3_) and 5 M of NH_4_F in the volume ratio of 1:1 for 30 s and a thin (~100 nm) layers of Ag nanoparticles were deposited on the surface of the wafers. In the second stage, c-Si wafers with Ag nanoparticles were placed in the etching solution containing 5 M of NH_4_F and 30% H_2_O_2_ in the volume ratio of 10:1 for 10 min. The PH value of the NH_4_F aqueous solution was changed by adding of H_2_SO_4_ droplets and varied in the range from 1 to 5. All the etching stages were carried out at room temperature. After the etching process all the samples were rinsed in de-ionized water and dried at room temperature. The main etching reaction the same that was described in Zhang et al. (2008):

(1) $$\begin{array}{l} \text{Si}+{\text{H}}_{2}{\text{O}}_{2}+{6\text{F}}^{-}+{4\text{H}}^{+}={\text{SiF}}_{6}^{2-}+{4\text{H}}_{2}\text{O}, \end{array}$$

however in our case the ions of *F*^−^ and *H*^+^ were obtained not from the dissociation of HF as in standard MACE procedure, but from the dissociation of NH_4_F and H_2_SO_4_. Ag nanoparticles played the role of catalysts for the etching process. The removal of Ag nanoparticals from SiNW arrays was performed by immersing in concentrated (65%) nitric acid (HNO_3_) for 15 min.

The structures of SiNWs were studied by a scanning electron microscope (SEM) of Carl Zeiss SUPRA 40 FE-SEM. Impedance spectra and Mott-Schottky measurements were performed using Solartron 1287 electrochemical interface and Solartron 1255B frequency response analyzer. All the measurements were carried out in three-electrode teflon cell using Ag/AgCl reference electrode joined through polypropylene Luggin capillary. The total reflectance (which includes both diffuse and specular components) spectra at the wavelength from 250 to 850 nm were studied with an integrating sphere on a Perkin Elmer spectrometer Lambda 950. The interband PL and Raman spectra were measured in a back scattering geometry with a Fourier-transform infrared (FTIR) spectrometer of Bruker IFS 66v/S equipped with a FRA-106 unit. Excitation was carried out by cw Nd:YAG laser at the wavelength 1.064 μm (excitation intensity was 100 mW and spot size was 2 mm). All experiments were carried out in air at room temperature.

## Results and Discussion

**Figure 1:**
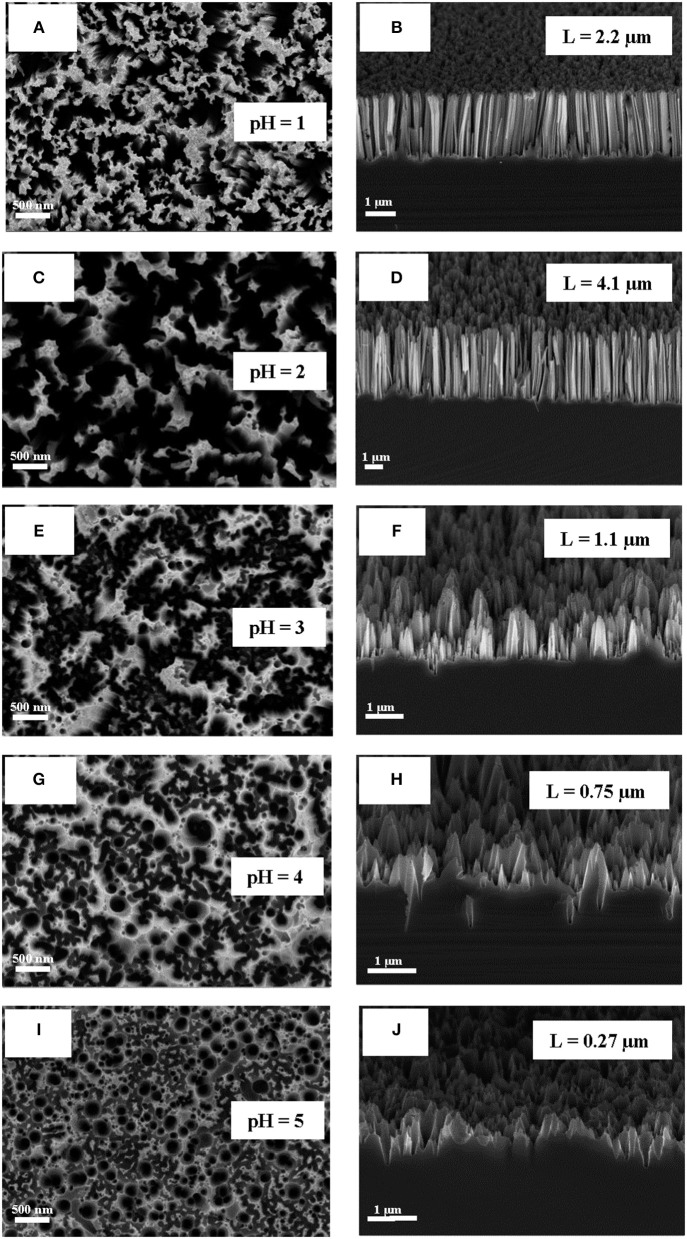
**(A,C,E,G,I)** SEM micrographs of SiNWs with different pH of H_2_O_2_:NH_4_F (view from above); **(B,D,F,H,J)** SEM cross-sectional micrographs of SiNWs with different pH of H_2_O_2_:NH_4_F.

**Figure 2:**
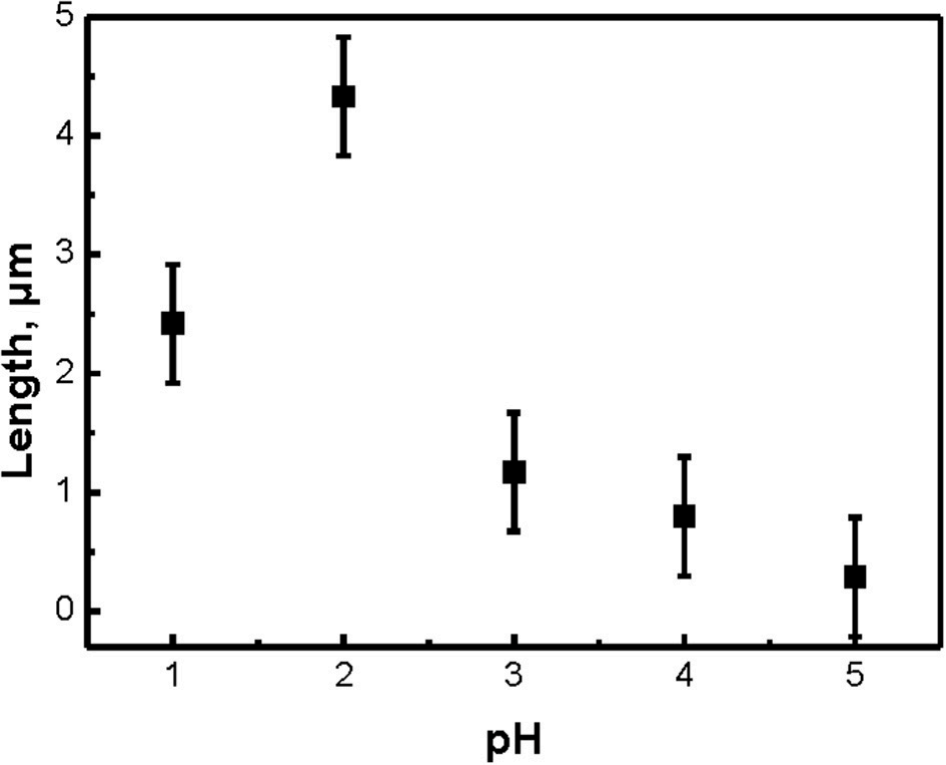
The dependence of the length of SiNWs with different pH value of H_2_O_2_:NH_4_F.

Typical SEM microphotographs of SiNW layers, which were obtained by using different pH of the etching solution H_2_O_2_:NH_4_F are presented in Figure 1. Note, that for pH = 6 or 7 the etching rate was very slow and the optical properties of SiNWs are slightly different from c-Si substrate. It is seen from the Figure 1, that the shape of SiNW is changing from vertical cylinders to pyramidal like structures with pH increasing. Figure 2 presented the dependence of the length of SiNWs from the pH value. The length of SiNW is maximum at pH = 2 and then decreases with increasing pH. SiNW porosity was calculated by using Bruggeman model (Bruggeman, 1935) and was approximately 50–60% for all samples.

**Figure 3:**
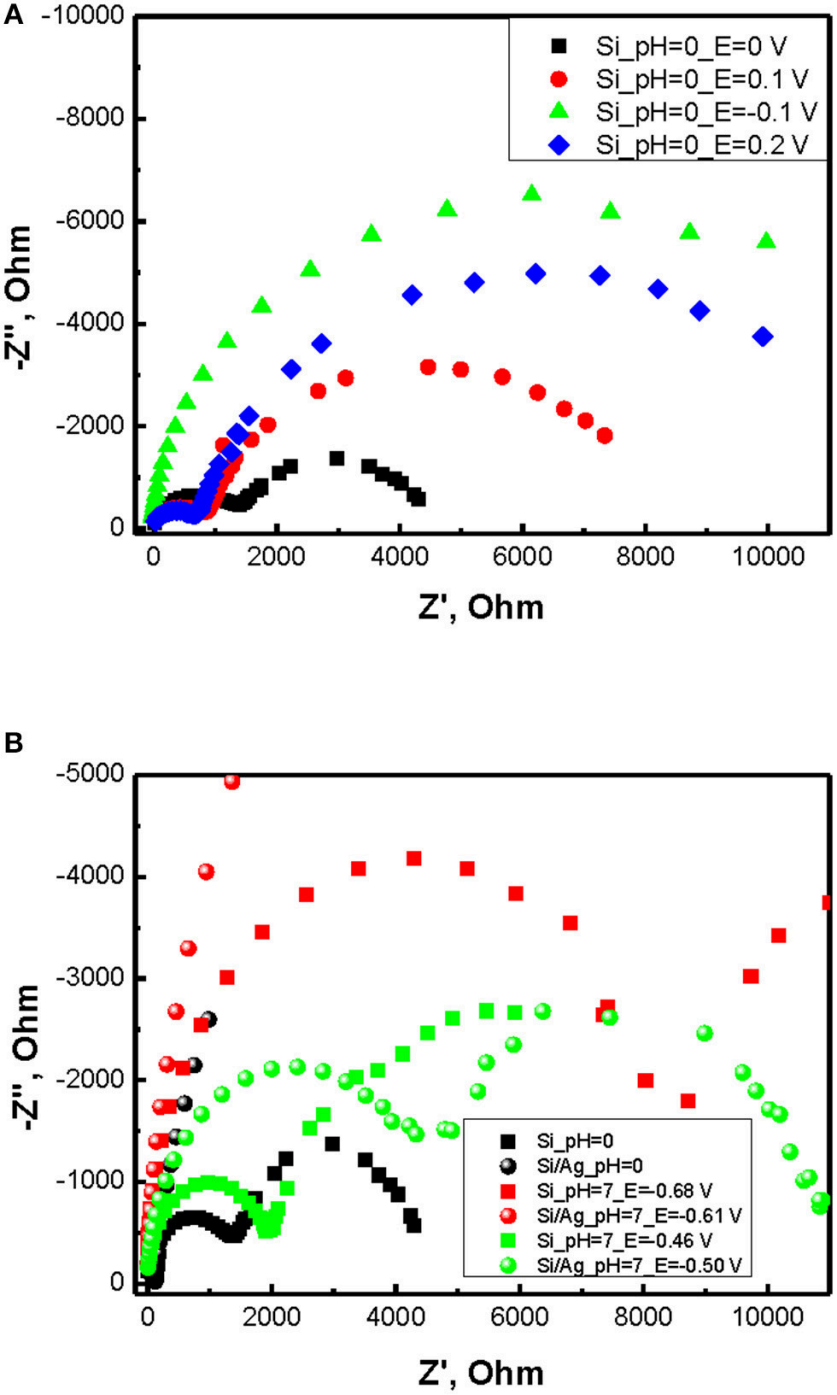
**(A)** Impedance spectra of p-doped silicon in ~5M NH4F/1M H_2_SO_4_ electrolyte containing 30% of H_2_O_2_. **(B)** impedance spectra for different interfaces and pH.

Impedance spectra of p-doped silicon in 5M NH_4_F/1M H_2_SO_4_ electrolyte containing 30% of H_2_O_2_ illustrate two semicircles with series resistance close to zero (Figure 3A). Thus, an equivalent circuit for the cell can be represented by parallel RC circuits connected in series. Applying positive bias vs. open circuit potential (OCP) leads to a first element resistivity decrease while increasing the radius of the second semicircle. Applying negative potential leads to first semicircle radius growth. As soon as Warburg resistance can be considered negligible in concentrated NH_4_F/H_2_SO_4_ solution, the presence of the second semicircle can be referred to an electric double layer with non-equilibrium silicon oxide formed at the surface of Si electrode. Resistivity of SiO_x_ layer predictably increases with shifting to positive potentials vs. Ag/AgCl reference due to growing layer thickness. As soon as first semicircle appear at higher frequencies (typically >1,000 Hz) it can only correspond to the processes at Si/SiO_x_ interface. This parallel RC element can be ascribed to the accumulation layer in Si resulting in downward bending of the valence and the conduction bands. Decreasing the radius of this semicircle with shifting to positive potentials is than well-explained by band flattening in p-doped silicon.

Notably both the SiO_x_ layer thickness and electrolyte potential are strongly affected by pH. With increasing pH of electrolyte OCP of the cell decreases reducing silicon oxidation rate (Figure 3B, Table 1). However, the radius of the second RC elements grows due to lower dissolution rate of silicon dioxide resulting in higher capacitance of the layer (Figure 3B).

**Table 1:** Open circuit potential (OCP) and flat band potential for different interfaces and pH.

**Sample**	**Open circuit potential, V**	**Flat band potential, V**
Si_pH = 0–1	−0.17	−0.26
Si_pH = 2–3	−0.25	−0.34
Si_pH = 4–5	−0.34	−0.46
Si_pH = 6–7	−0.55	−0.75
Si/Ag_pH = 0–1	−0.14	−0.32
Si/Ag_pH = 2–3	−0.30	−0.38
Si/Ag_pH = 4–5	−0.33	−0.39
Si/Ag_pH = 6–7	−0.42	−0.50

Addition of silver particles to the system introduces a number of changes to the impedance spectra. First, Z” at high frequencies strongly decreases implying lowering of the capacitance at Si/SiO_x_ interface. Secondly, the radius of the second semicircle greatly increases indicating larger thickness of SiO_x_ layer. These effects are associated with inhomogeneous nature of Ag/Si electrodes where both silver coated and uncoated regions contribute the impedance spectra. Probably Si/SiO_x_/Ag/Ag_2_O/H_2_O_2_ electrochemical chain provides smaller barrier as compared to direct electric double layer contact Si/SiO_x_/H_2_O_2_. However, in case of low frequencies the depletion of charge carriers from Ag/Si results in limitation of carrier transport and SiO_x_ layer capacitance growth.

**Figure 4:**
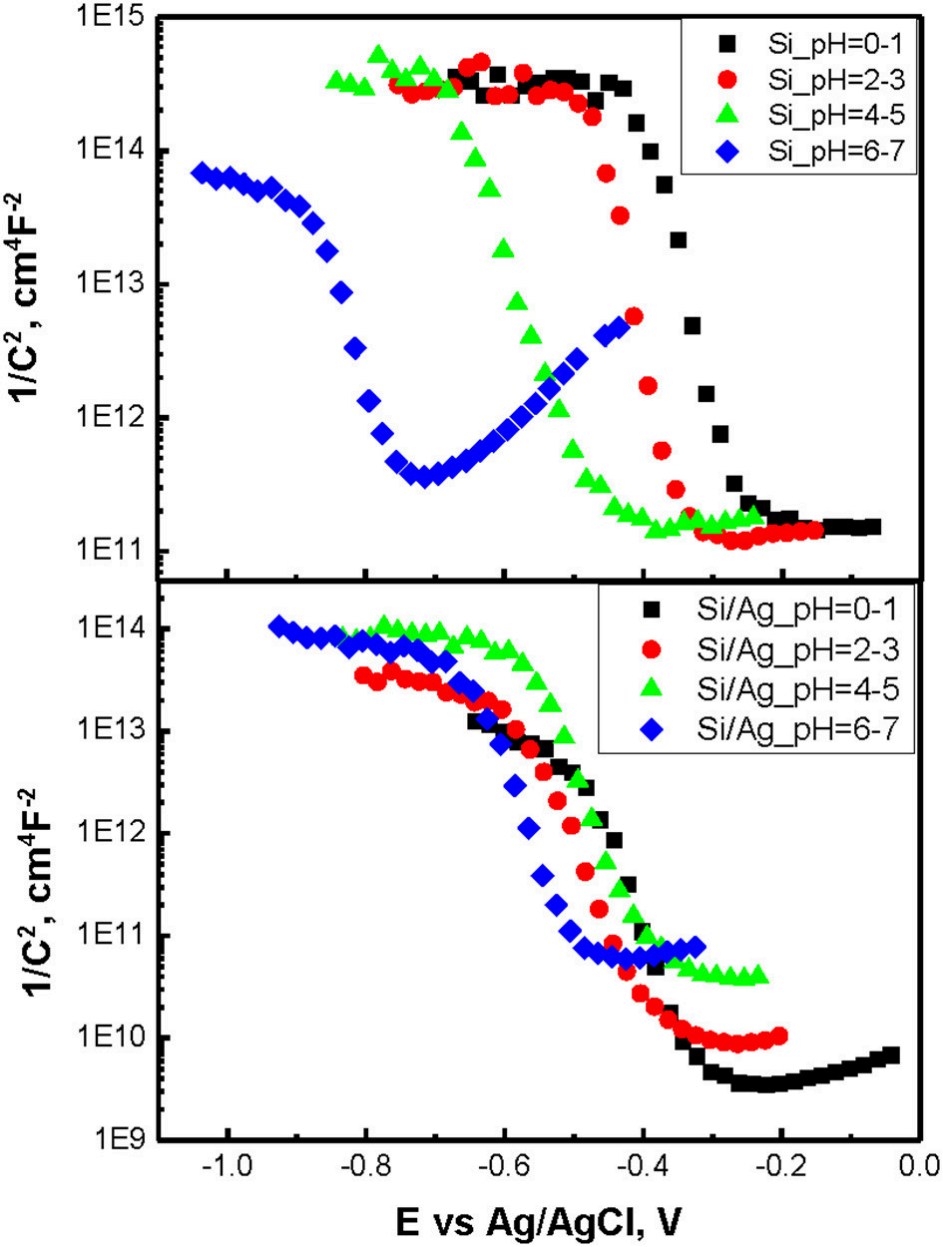
Mott-Schottky measurements at 1,000 Hz.

To determine flat band potentials of p-doped Si and Ag/Si electrodes Mott-Schottky measurements were performed at 1,000 Hz. The choice of the frequency was dictated by the necessity to attain depletion of the charge carriers while avoiding diffusion limitations. Resulting plots for different pH of etching solutions and derived flat band potential values are summarized in Figure 4 and Table 1. One can see, the flat band potentials being pH dependent in case of etching of p-doped silicon converge into closely the same value in case of Ag/Si. On the other hand, OCP values stay very close in both p-Si and Ag/Si, with only small shift of OCP in case of Ag/Si. This effect corresponds well to smaller band bending and smaller capacitance of the interface layers. Thus, silver assisted chemical etching of silicon can be ascribed to facilitated transport through Si/SiO_x_/Ag interface.

**Figure 5:**
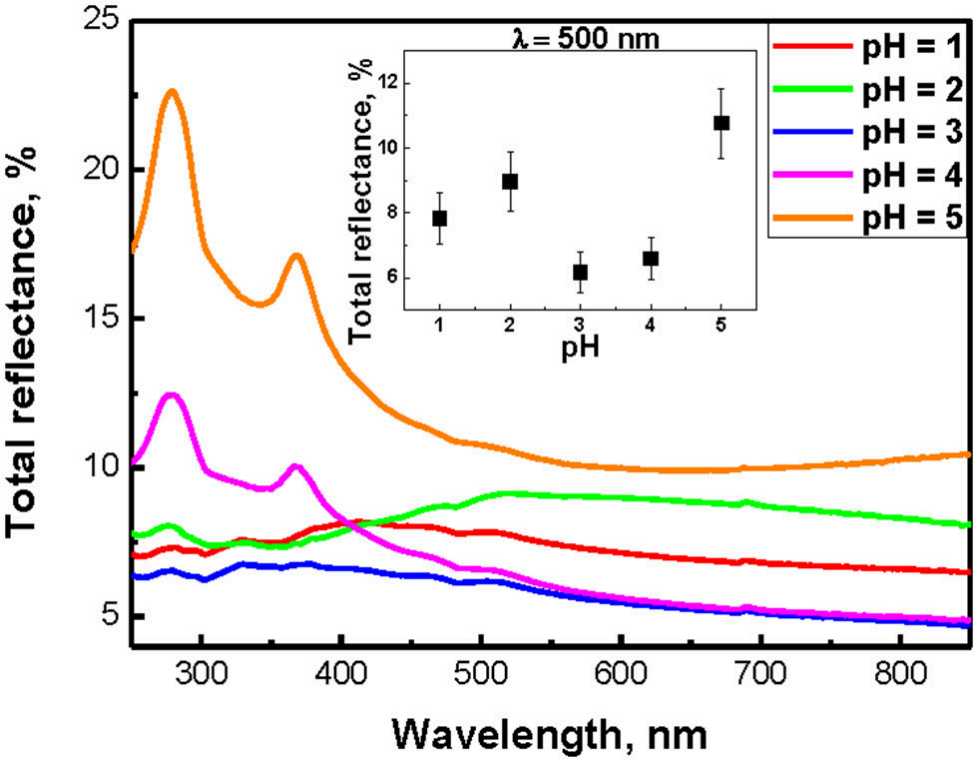
Total reflectance spectra of SiNWs with different pH of H_2_O_2_:NH_4_F; inset shows the dependence of total reflection of SiNWs from the pH value of H_2_O_2_:NH_4_F.

Total reflectance spectra of SiNW layers are presented in Figure 5. All samples exhibit a strong decrease of the total reflectance to 5–10% at the wavelength <850 nm in comparison to c-Si substrate (50%). At pH >3, the total reflection spectra of nanowires have a very similar form with c-Si, since for a weak submicron length SiNW, the c-Si substrate has a significant effect on the reflection value. Also in this case, reflection peaks appear at 280 and 370 nm, which are associated with the c-Si direct band gap. Low total reflection of SiNW layers can be explained by the strong scattering and absorption of light in the visible region of the spectrum, which can lead to a partial localization of light in nanowires (Gonchar et al., 2012). The inset in Figure 5 shown the dependence of the total reflection of SiNWs at 500 nm from the pH value of H_2_O_2_:NH_4_F. It is seen that for this wavelength, all samples have the same low values of the total reflectance (5–10%).

**Figure 6:**
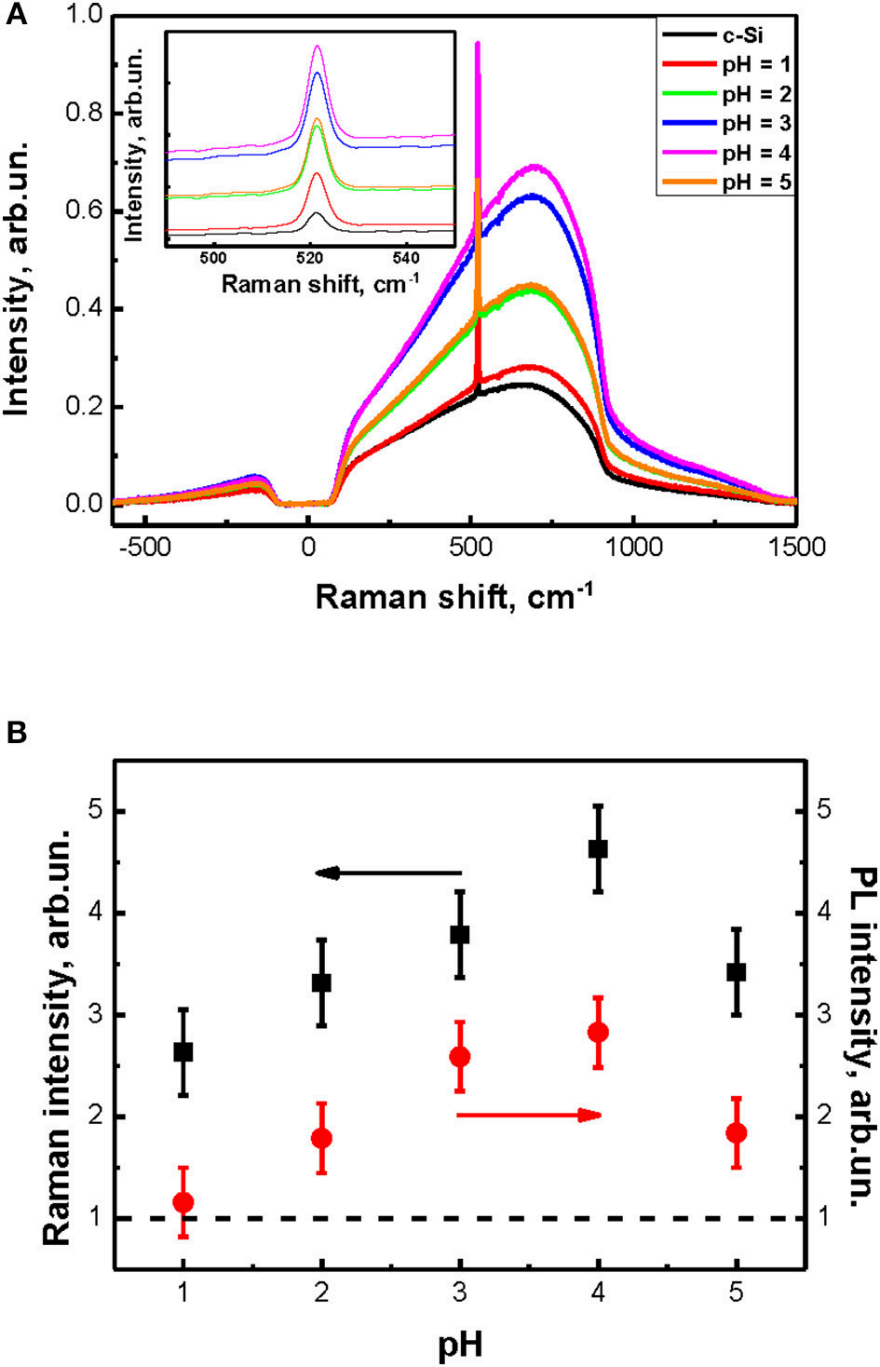
**(A)** Spectra of interband PL and Raman scattering of c-Si substrate and SiNWs with different pH of H_2_O_2_:NH_4_F, inset shows Raman scattering peaks of c-Si substrate and SiNWs with different pH of H_2_O_2_:NH_4_F. **(B)** The dependence of intensities of Raman scattering and interband PL of SiNWs from the pH value of H_2_O_2_:NH_4_F.

The spectra of interband PL (broad peak) and Raman scattering (sharp peak at 520 cm^−1^) of the c-Si substrate and a number of SiNW grown at different pH values are shown in Figure 6A. The inset in Figure 6A shows a close view of the Raman scattering peaks. SiNW's diameter is about 50–200 nm and far from the quantum confinement regime. That's why peaks and shapes of the interband PL and Raman scattering for all samples are similar to c-Si. At the same time the intensities of interband PL and Raman scattering for SiNWs increase strongly as opposed to corresponding value for c-Si. This effect can be explained by the light localization in such inhomogeneous optical medium as SiNW layers (Gonchar et al., 2014).

Figure 6B shows the calculated from Figure 6A dependence of SiNW's Raman scattering and interband PL intensities from the pH value. The signal intensity of the samples here was normalized to the signal intensity of c-Si substrate (dash line). Thus, the intensity of Raman scattering and interband PL increases by 3–5 times and 3 times, respectively, for all SiNWs layers in comparison with c-Si. Let's remember, that the shape and length of SiNWs is changed with the increasing of pH value of H_2_O_2_:NH_4_F: the length is decrease and the shape is changing from vertical cilinders to pyramidal like structures (see Figure 1. Based on this, we can conclude that the intensity of Raman scattering and interband PL depends not only on the length of SiNW, but also on their shape.

## Conclusion

The structural and optical properties of SiNWs, prepared by the metal assisted chemical etching method, where the commonly used hydrofluoric acid (HF) has been successfully replaced with ammonium fluoride (NH_4_F), and their dependence from the pH of the etching H_2_O_2_:NH_4_F solutions were studied in detail for the first time. It is shown that as the pH of H_2_O_2_:NH_4_F decrease, the shape of the nanowires changes from pyramidal to vertical. The length of SiNW arrays demonstrated non-linearly pH dependence. By impedance and Mott-Schottky measurements it was shown that the SiO_x_ layer thickness and electrolyte potential are strongly affected by pH. With increasing pH of electrolyte OCP of the cell decreases reducing silicon oxidation rate. Silver assisted chemical etching of silicon can be ascribed to facilitated charge carriers transport through Si/SiO_x_/Ag interface. All samples exhibit a strong decrease of the total reflectance to 5–10% at the wavelength <800 nm in comparison to c-Si substrate. Also the intensities of interband PL and Raman scattering for SiNWs increase strongly as opposed to corresponding value for c-Si, but depends both from the length and the shape of SiNWs: they were larger for long pyramidal nanowires. This effect can be explained by the light localization in such inhomogeneous optical medium as SiNW layers Thus, SiNW, manufactured using weakly toxic NH_4_F, have great potential for applications in the field of photovoltaics, photonics, and sensorics.

## Author Contributions

KG and VK performed SiNWs fabrication, optical measurements, and data analysis. GZ performed the SEM measurements. AE performed impedance and Mott-Schottky measurements. KG and LO performed the general data analysis and discussion of the obtained data. All authors read and approved the final manuscript.

### Conflict of Interest Statement

The authors declare that the research was conducted in the absence of any commercial or financial relationships that could be construed as a potential conflict of interest.
